# Atypical Hepatic Hemangioma With Progressive Centrifugal Enhancement Pattern Mimicking Malignant Liver Lesion

**DOI:** 10.7759/cureus.39901

**Published:** 2023-06-03

**Authors:** Tom Mishael J, Arun George, Babu Philip, Sandeep S

**Affiliations:** 1 Radiology, St. John's Medical College Hospital, Bangalore, IND

**Keywords:** malignancy, ct, centrifugal enhancement, atypical hepatic hemangioma, hepatic hemangioma

## Abstract

Hepatic hemangioma is a common benign vascular hepatic lesion with typical imaging features. However, hepatic hemangiomas with atypical radiological characteristics can sometimes be diagnostically challenging. Here, we report a case of an elderly patient with colonic adenocarcinoma who was incidentally found to have an atypical hepatic hemangioma showing a progressive centrifugal enhancement pattern instead of a typical centripetal pattern on contrast-enhanced computed tomography mimicking a malignant liver lesion.

## Introduction

Hepatic hemangioma, or hepatic venous malformation, is the most common benign vascular liver lesion with well-established imaging characteristics. A typical hepatic hemangioma is a well-defined hyperechoic lesion on ultrasound which is a hypoattenuating lesion on computed tomography (CT) showing discontinuous, nodular, peripheral enhancement on the arterial phase with progressive centripetal fill-in on the portal venous, venous, and delayed phases [1]. However, some unusual imaging patterns, such as hepatic hemangiomas with capsular retraction, surrounding regional nodular hyperplasia, fatty infiltration, cystic changes, fluid-fluid levels, peduncles, and calcifications, have also been reported [2]. We report a case of an elderly patient with a hepatic hemangioma showing a centrifugal enhancement pattern on dynamic contrast-enhanced CT mimicking malignant liver lesions.

## Case presentation

**Figure 1:**
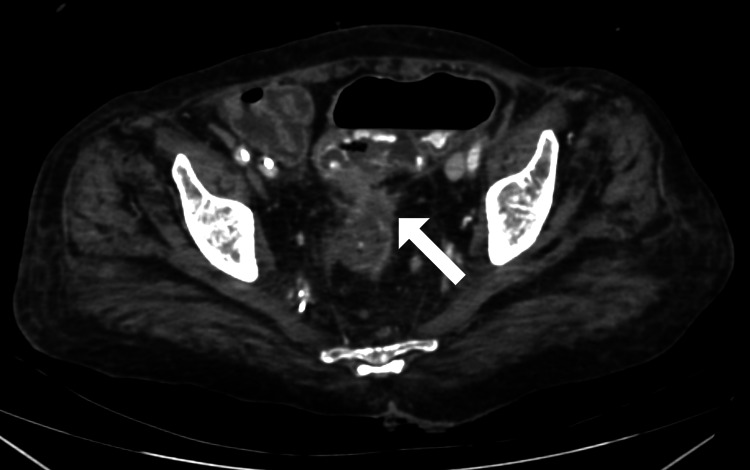
Axial post-contrast CT image of the abdomen showing irregular mural thickening and luminal narrowing involving the rectosigmoid junction

**Figure 2:**
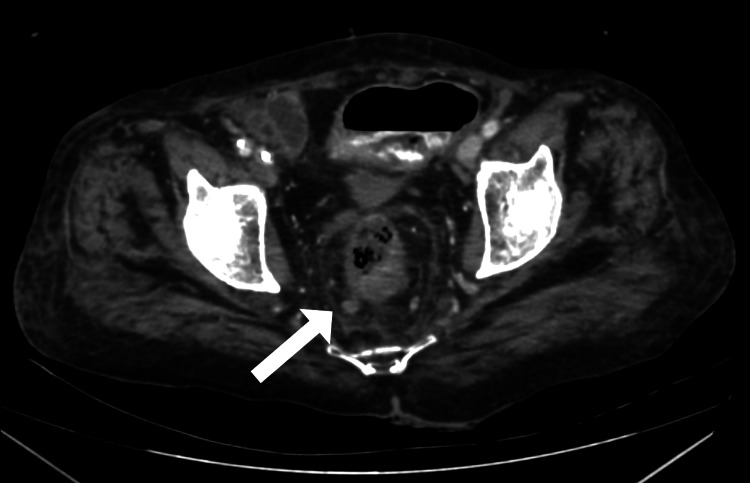
Axial post-contrast CT image of the abdomen showing thickening of the mesorectal fascia, regional fat stranding, and perirectal lymphadenopathy around the mural thickening in the rectosigmoid junction

A 65-year-old male who presented with decreased appetite, lethargy, significant weight loss, and hematochezia was found to have iron deficiency anemia and was advised to undergo a contrast-enhanced CT scan of the abdomen. The blood hemoglobin level of the patient was 8.6 g/dL at the time of presentation. However, he was hemodynamically stable. His liver function tests had normal findings, and his tests for viral markers (hepatitis B and C) were negative. After analyzing the pre-contrast images, the radiology team advised a triphasic contrast study of the abdomen after giving neutral oral and rectal contrast. On post-contrast images, irregular mural thickening and luminal narrowing involving the rectosigmoid junction (Figure 1), associated with thickening of the mesorectal fascia, regional fat stranding, and perirectal lymphadenopathy (Figure 2), were seen.

**Figure 3:**
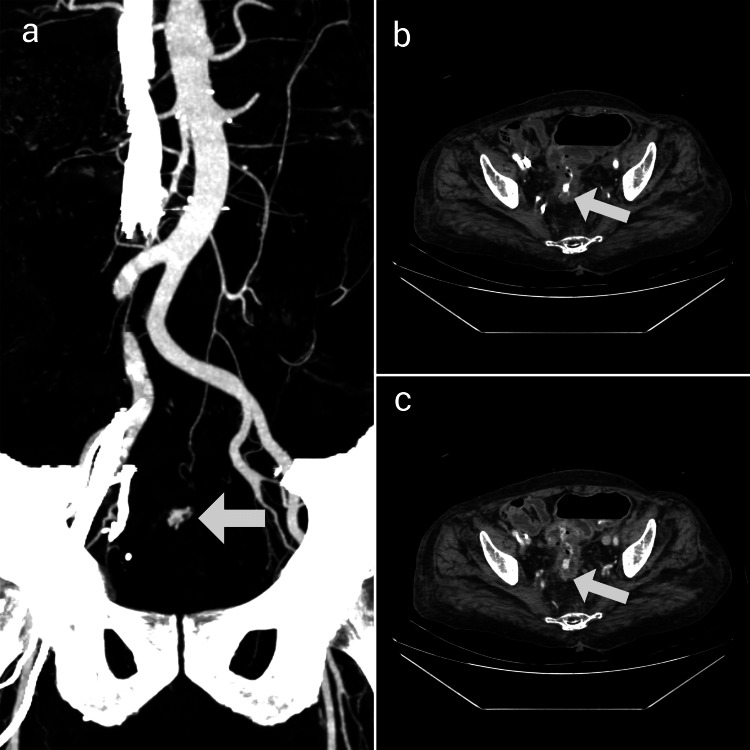
Coronal Maximum Intensity Projection (MIP) image (a) and axial post-contrast arterial phase (b) and venous phase (c) images showing active extravasation of contrast from a prominent branch of the superior rectal artery associated with pooling of contrast within the lumen of the sigmoid colon

There was active contrast extravasation into the bowel lumen at the same site from a prominent branch of the superior rectal artery associated with the progressive pooling of contrast material within the lumen of the sigmoid colon (Figure 3). However, there was no evidence of intestinal obstruction or perforation. The aforementioned features were of concern for colonic malignancy.

**Figure 4:**
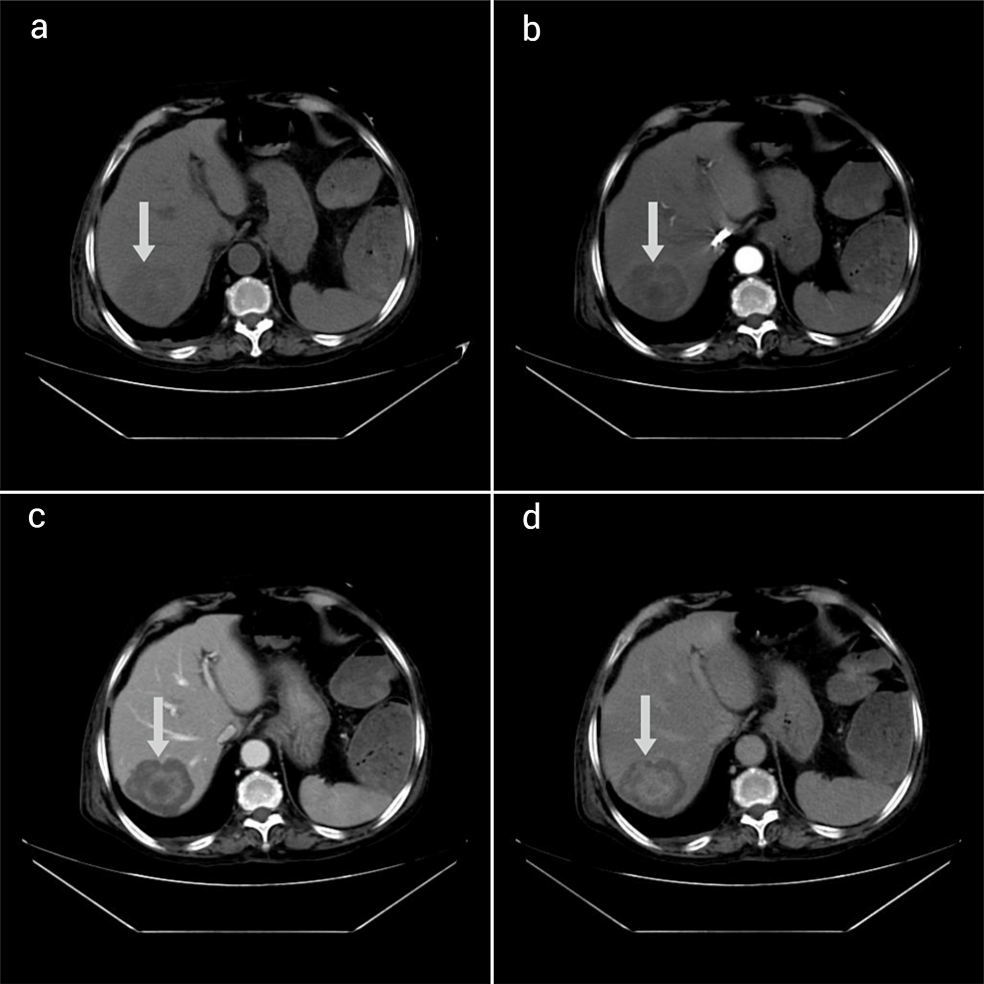
Axial image of non-contrast CT of the upper abdomen (a) shows a well-defined hypodense lesion in segment VI of the liver. On post-contrast axial images, the lesion shows central arterial phase hyper-enhancing areas (b) with progressive centrifugal enhancement in portal venous (c) and delayed phase (d) images.

A well-defined, hypodense lesion measuring 4.8x4.6cm and showing central hyper-enhancing areas on arterial phase images with a progressive, centrifugal, or inside-out pattern of enhancement on portal venous, venous, and delayed phase images was noted in segment VI of the liver (Figure 4).

A peripheral, unenhanced portion was still visible on five-minute delayed images. There was no evidence of dilatation of the common bile duct or intra-hepatic biliary radicles. No other differentially enhanced lesions were found in the liver. Although the imaging findings were atypical, considering the age and clinical features of the patient, hepatic metastasis from a malignant colonic neoplasm was given as the most likely diagnosis. However, an alternate possibility of hepatic hemangioma with an atypical enhancing pattern was also suggested.

**Figure 5:**
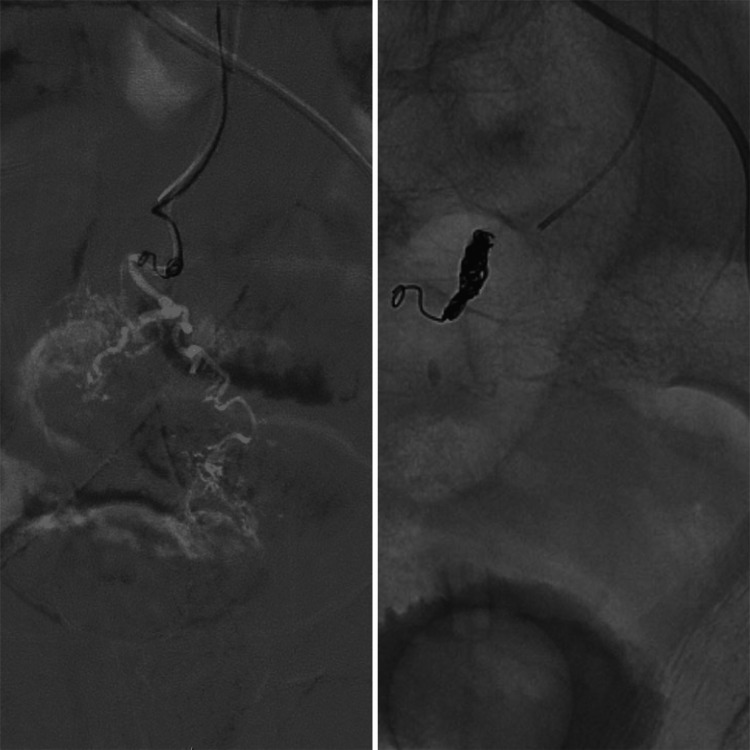
Digital subtraction angiography images showing selective catheterization and endovascular coil-embolization of the actively bleeding branch of the superior rectal artery

The interventional radiology team took the patient for digital subtraction angiography. Endovascular intervention was preferred over endoscopic examination since the patient had active hemorrhage from a prominent and tortuous branch of the superior rectal artery and had not undergone bowel preparation. Selective catheterization and endovascular coil embolization of the actively bleeding branch of the superior rectal artery were performed (Figure 5) by the interventional radiology team. The patient tolerated the procedure well, and there was no further drop in blood hemoglobin level.

On colonoscopic biopsy of the rectosigmoid lesion, the lesion was histopathologically proven to be an adenocarcinoma colon. The treating team also decided to go ahead with an ultrasound-guided biopsy of the liver lesion after taking high-risk consent from the patient, in view of atypical imaging features, in order to obtain a definitive diagnosis. The patient tolerated the procedure well. However, the histopathological diagnosis was surprisingly suggestive of a cavernous hemangioma of the liver.

The patient was treated by transabdominal resection of the colonic carcinoma, followed by adjuvant chemotherapy and radiotherapy. The patient tolerated the treatment well. On follow-up after one month after discharge from the hospital, the patient did not have any new complaints, and the blood hemoglobin level improved to a value of 10.9 g/dL.

## Discussion

Large vascular spaces with thin intervening septa were observed in hepatic hemangiomas that had rapid arterial phase enhancement, as opposed to smaller vascular components and a significant amount of fibrous interstitial space in hemangiomas that had delayed enhancement. It is likely that puddling, poor blood flow, and partial thrombosis are the causes of prolonged retention of contrast in vast intravascular spaces, which leads to delayed contrast enhancement in hepatic hemangiomas [3].

Fibrosis of hepatic hemangioma often begins in the lesion's center and spreads peripherally to varying degrees [4]. However, the histopathological findings of the patient showed a predominance of fibrous tissue in the periphery and numerous vascular spaces in the center. This explains why the lesion showed early central hyper-enhancement and delayed peripheral enhancement on dynamic contrast-enhanced CT images.

A few authors have described atypical hepatic hemangiomas with a centrifugal or inside-out pattern of enhancement [5]. However, the simultaneous occurrence of such a hepatic lesion and a primary malignancy in an elderly patient has not been described in the literature before. Atypical imaging findings in this patient were likely caused by the variation in structural characteristics of the hepatic hemangioma.

## Conclusions

Most hepatic hemangiomas have typical radiological features that are extremely reliable and well-studied. However, intra-tumoral anatomical and physiological variations can result in atypical imaging features. Confusing clinical histories and co-existing diseases can make imaging diagnoses even more challenging. Histopathological confirmation may be necessary in such rare scenarios to draw further conclusions.
